# Three-Dimensional (3D) Culture Models in Cancer Investigation, Drug Testing and Immune Response Evaluation

**DOI:** 10.3390/ijms22010150

**Published:** 2020-12-25

**Authors:** Roberto Benelli, Maria Raffaella Zocchi, Alessandro Poggi

**Affiliations:** 1 Unit of MolecularOncology and Angiogenesis, IRCCS Ospedale Policlinico San Martino, 16132 Genoa, Italy; roberto.benelli@hsanmartino.it; 2 Division of Immunology, Transplants and Infectious Diseases, IRCCS Scientific Institute San Raffaele, 20132 Milan, Italy; zocchi.maria@hsr.it

Preclinical models for the definition of anti-cancer drug safety and efficacy are constantly evolving. Indeed, tumor development in humans is not always fully reproducible and predictable in other animals. In turn, two-dimensional (2D) cultures, used for many years to test drug effects, are limited by the lack of tissue structure and architecture, which can influence both pharmacokinetics and pharmacodynamics, thus impairing the prediction of anticancer drug efficacy. An interesting and reliable alternative is represented by three-dimensional (3D) culture systems, including spheroids and matrix-based collagen or synthetic scaffolds, which have been validated by the EU Reference Laboratories as preclinical models to overcome at least some of the above-mentioned drawbacks.

This thematic issue collects studies involving both advances in the techniques used to create and analyze some 3D culture systems and their applications in different cancer models for drug combination testing. The switch from 2D to 3D culture changes several conditions for cell growth, the first being a reduced surface for direct targeting, with the inner cell layers being protected from the external environment. At the same time, inner cells can suffer a reduced nutrient or oxygen supply, drastically changing their metabolism to a hypoxic one, causing the transcription of genes typically involved in drug resistance. This picture is even more complicated when co-cultures of different cell types are generated.

A. R. Holub et al. [1] used an ultra-high resolution printer to generate large basket scaffolds (2000μm), allowing oxygen permeation, eventually populated with MDA-MB-231 and MCF-7 breast cancer cell lines. This model was compared to small self-aggregated spheroids to test different formulations of Doxorubicin (Doxo), finding Doxo-charged nanoparticles to be the most effective in both models. The authors underlined both the advantages of using large basket scaffolds (rapid formation of spheroids, formation of regular structures using cells that do not organize spontaneously) and their possible limitations (large structures resist light penetration for microscopic analysis), suggesting that further studies are needed to evaluate this scaffold under dynamic conditions and in co-culture models.

The bioprinting approach tested by Seokgyu Han et al. [2] allowed the creation of a complex multicellular scaffold mimicking the tumor microenvironment. An endothelial-fibroblast mix glued with alginate, gelatin and fibrinogen hydrogel was used to generate 3D vascular-like networks in culture. These structures were eventually seeded with U87-derived glioblastoma spheroids and the neovascularization of spheroids and tumor sprouting were monitored, showing the interactions of the different cellular components. U87 spheroids cultured in bioprinted matrix and enriched in vascular structure showed increased expression of the epithelial–mesenchymal transition markers N-cadherin and vimentin, indicating a cross-influence among tumor and normal cells. This model was treated with the anticancer drug temozolomide and the anti-angiogenic molecule sunitinib, showing the synergism of their combination to affect both tumor spheroid size and the vascular network.

Ezequiel Monferrer et al. [3] compared the behavior of SK-N-BE and SH-SY5Y neuroblastoma cell lines in gelatin-based scaffolds with different stiffness with or without the addition of the stromal Schwann cell line SW10. Each cell line showed a different modulation of analyzed markers (Ki67, vitronectin, DOCK8 and KANK1) and different behavior according to stiffness and co-culture condition, indicating specific signatures that could be used to define different tumor cell models and providing a strategy to contrast their growth.

Although these models of scalable complexity allow us to investigate the interactions between tumor spheroids and the tissue microenvironment, their analysis is usually confined to classic microscopy approaches. Optical microscopy shows intrinsic limits that can be usually outdone only by high cost instruments. However, Eliana Steinberg et al. [4] have demonstrated a simple and inexpensive protocol to highly maximize the resolution of 3D microscopy up to the 200-micron depth with standard confocal equipment. In their model, high-density pancreatic spheroids were characterized, assessing their organization, metabolism, interaction with stromal and endothelial cells, dynamic morphological changes and nanoparticle permeation. This clarifying method surpasses the standard methods limiting the unbiased detection of fluorescent signals through the first 50µm of spheroid surfaces, while allowing the characterization of inner cell layers. This increased depth allowed the authors to show that endothelial cells are recruited to the inner part of pancreatic spheroids, corresponding to the expected hypoxic region, without spheroid dissection.

Another approach to increasing the information derived from 3D models has been described by Tarapong Srisongkram et al. [5], applying Fourier transform infrared (FTIR) microspectroscopy to the SK-MEL-2 melanoma model. The absorbance spectra of FTIR can be used to quantify basic cellular components: lipids, proteins, DNA and RNA. Melanoma cells showed a marked shift when analyzed in 2D or 3D culture systems—3D cultures showed lower lipid, DNA and RNA levels and increased protein content compared to 2D cultures. Moreover alpha-helix-organized proteins prevailed in 2D culture, whereas beta-turns prevailed in 3D culture. This approach was also able to quantify the extent of necrosis in the center of spheroids, showing that aggregates of 20,000 cells were particularly susceptible to necrosis and showed a different FTIR shift, as compared to smaller ones. These large structures also showed significant changes in protein secondary structures. Thus, FTIR is a valid method to monitor spheroids at the molecular level.

Although new techniques allow researchers to extract an increasing amount of data from 3D cultured cells, the more immediate application of these models is still drug testing. This goal was pursued by Sadaf E. Pustchi et al. [6], testing the influence of human normal astrocytes on the behavior of glioblastoma multiforme (LN299 cell line) under therapy in a 3D model. Temozolomide and the NF-κB inhibitor Bay11-7082 were tested alone and in combination. Both LN299 and normal astrocytes cultured alone showed susceptibility to these drugs, with minimal differences if used alone or in combination. On the contrary, co-cultures showed increased resistance to drugs and a significant increase in efficacy by their combination. In this condition, TUNEL analysis indicated that LN299 cells were the principal drug targets; indeed, GFAP and vimentin were selectively downregulated in these cells, whereas other markers varied in both cell populations.

A combined therapeutic regimen was also investigated by Layla Mohammad Hadi et al. [7] in 2D and 3D ovarian cancer cell cultures. Photodynamic therapy and dactinomycin were tested at sub-lethal doses, showing a slightly higher efficacy in 2D systems compared to 3D systems. The efficacy of photodynamic therapy was also dependent on the time of irradiation. The use of compressed collagen scaffolds allowed the authors to mimic the high density of the tissue matrix, in which oxygen (necessary for photodynamic therapy) has a reduced ability to diffuse. Accordingly, this model could reproduce a more physiological situation in which to test these drug associations. Last but not least, Fabrizio Fontana et al. [8] reviewed 3D culture methods and applications, focusing on prostate cancer. In their paper, multiple approaches to 3D cultures, characterization and experimental use are reported to obtain a comprehensive view of a field in active and continuous development.

In summary, 3D cultures have contributed deeply to improving the reliability of in vitro pre-clinical models in anti-cancer drug testing. Moreover, with the establishment of organoids from various types of epithelial cancers, co-cultures of cancer organoids and immune cells have become a highly informative strategy for the development and testing of cancer immunotherapy, representing an additional useful application of 3D culture systems in cancer treatment.
